# Evaluation of egg production after adoption of biosecurity strategies by backyard poultry farmers in West Bengal

**DOI:** 10.14202/vetworld.2015.177-182

**Published:** 2015-02-16

**Authors:** I. Samanta, S. N. Joardar, D. Ganguli, P. K. Das, U. Sarkar

**Affiliations:** 1 Department of Veterinary Microbiology, West Bengal University of Animal & Fishery Sciences, Belgachia, Kolkata, West Bengal, India; 2 Department of Veterinary and Animal Husbandry Extension Education, West Bengal University of Animal & Fishery Sciences, Belgachia, Kolkata, West Bengal, India; 3 Department of Veterinary Physiology, West Bengal University of Animal & Fishery Sciences, Belgachia, Kolkata, West Bengal, India; 4 Department of Animal Genetics and Breeding, West Bengal University of Animal & Fishery Sciences, Belgachia, Kolkata, West Bengal, India

**Keywords:** adoption, backyard poultry, biosecurity strategy, egg production, West Bengal

## Abstract

**Aim:**

On the basis of identified source of major bacterial infections at four agro-climatic zones in West Bengal the cost-effective biosecurity strategy was formulated for backyard poultry farmers. The aim of the present study was to assess the adoption. So, the study was aimed to detect the adoption level of the formulated biosecurity strategy to mitigate the *Salmonella* and *Escherichia coli*week post-hatch period chicks were contamination level in the sources and its correlation with egg production in West Bengal.

**Materials and Methods:**

A questionnaire was prepared querying regarding the biosecurity measures presently followed by the farmers, if any and egg production of their birds. Subsequent to the interview the formulated biosecurity strategy was conveyed. After 3 months, the interview with the same questionnaire was conducted to the same farmers to detect their adoption level.

**Results:**

The change in practices were noted in certain parameters which differs significantly (p<0.01 or p<0.05). As a consequence, the average egg production/flock was increased in 3 months after adoption of the strategy (618.2±37.77/flock) in comparison to last 3 months average before adoption of the strategy (495.3±30.00/flock) which also differs significantly (p<0.01).

**Conclusion:**

The present study detected the implementation of the biosecurity strategy in backyard poultry farming in West Bengal can substantially benefit the farmers in terms of increased egg production.

## Introduction

In developing countries such as India adoption of intensive poultry production system is limited due to the need for high inputs and resources. In rural and peri-urban areas access to poultry meat and eggs depends on backyard production system. In India, backyard poultry farming provides valuable protein through a low input system, now representing 30% or more of all protein consumed [1]. However, such backyard flock make a very minor contribution to rural livelihoods, as the income per bird per month ranges from Indian Rupees 4-13 with an average flock size of 5-20 in a household [2]. So the backyard farming does not appear to be a promising strategy to achieve the poverty reduction until the production level is increased [3]. There are some constraints in increasing the egg production in backyard birds such as microbial infection due to lack of biosecurity knowledge among the farmers [4].

Biosecurity is a set of preventive measures designed to reduce the risk of transmission of infectious diseases specially in organized poultry ­sector ­throughout the world [5]. Effective biosecurity measures to control *Salmonella* in commercial poultry include all-in-all-out system followed by disinfection of sheds, restricted movement of birds, workers and equipments, lack of contact with migratory birds, use of feed pellets, chlorination of drinking water, proper litter management, washing and sanitizing of hatching eggs and purification of air in hatching cabinets [6]. However, the studies related with farm level cost of these biosecurity measures are limited. Recently a study conducted in a broiler farm in Finland detected 3.55 Euro cents (Indian Rupees 2.75) per bird as average biosecurity cost which may be reduced further with increased numbers of the birds [7]. Further, implementation of full site biosecurity in backyard farming system is rather difficult than the organized sector due to lack of awareness, cost-effective measures and its correlation with the economic benefit as observed in different developing countries [8-11].

The study was conducted in West Bengal, where about 81% of the eggs are produced in backyard farming system. However, the state stands fifth in total egg production in India due to presence of low yielding backyard poultry population which is major egg producers [12]. Microbial infection in backyard chickens is major constraint in increasing the egg production which occurs due to lack of biosecurity measures throughout West Bengal.

So, the study was aimed to detect the adoption level of the formulated biosecurity strategy to mitigate the *Salmonella* and *Escherichia coli* contamination level in the sources and its correlation with egg production in West Bengal.

## Materials and Methods

### Ethical approval

The study was approved by Institutional Biosafety Committee, West Bengal University of Animal and Fishery Sciences, Belgachia, Kolkata.

### Formulation of biosecurity strategy

**Figure-1:**
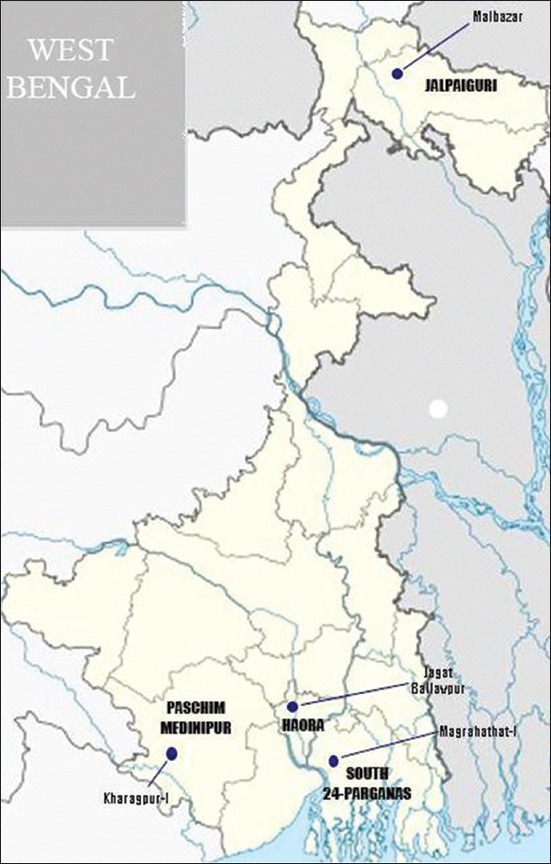
Locations of the study area in West Bengal

The study area included the villages from Mal block, Jalpaiguri district (terai zone); Jagatballavpur block, Howrah district (new alluvial zone); Kharagpur-I block, West Midnapur district (red laterite zone) and Magrahat-I block, South 24 Parganas district (coastal zone) (Figure-1). Each of the blocks in the study area maintained 100-300 backyard flocks comprised of 5-25 birds in each flock. The average minimum and maximum temperatures and rainfall during the sample collection period were 22°C and 31°C and 8 mm, respectively, in terai zone, 27°C and 34°C and 5 mm, respectively, in red latterite zone, 26°C and 33°C and 7 mm, respectively, in the new alluvial and costal zones. The studied birds (Rhode Island Red breed) were maintained in a semi-scavenging system which roamed around the farmer’s house during daytime and took shelter in a poultry house made of bamboo with asbestos shed. The source of bacterial infection (*Salmonella* and *E. coli*) such as feed, drinking water in the backyard birds in different studied agro-climatic zones (terai, new alluvial, red latterite, coastal) was identified [13,14]. Accordingly, the following ten-point biosecurity strategy was formulated to mitigate the *Salmonella* and *E. coli* contamination level in the sources.

a) The feed mixture offered to the birds should be washed with boiled water
b) The potable drinking water preferably boiled should be provided to the birds
c) The utensils for the feed or drinking water should be cleaned with detergent/ash daily
d) The drinking water in the utensil should be changed daily
e) The litter in the poultry house/dried manure under the house should be changed/cleaned frequently
f) The scavenging area of the birds during daytime should be restricted within the house premises. The detergent water (remaining water after cloth washing) may be sprinkled daily in their scavenging area
g) The entry of the wild birds and rodents should be restricted in the house premises
h) Travelling of the farmers to broiler/layer poultry farm or market should be restricted. If travelled, the intruder should enter the poultry house after proper cleaning himself and his dress materials
i) The carcass of the birds should be disposed of through proper way, preferably by garden burial
j) The eggs should be washed with sterile water and preserved at cooling temperature. If the refrigerator is not available, eggs can be stored in a clay pot with a wet cover. The cover should be removed, dipped into water and again placed in the mouth of the pot 5-6 times a day in summer and 2-3 times a day in winter.

### Assessment of adoption of the biosecurity strategy

An agro-climatic zone (terai) was selected for assessment of adoption of the formulated biosecurity strategy. A questionnaire was prepared querying regarding the biosecurity measures presently followed by the farmers and egg production by the birds. The questionnaire was pilot tested on 3 flock owners before going to the field. The ‘owner’ was defined as the person normally in charge of the flock who were generally female members of the households in the studied area. The flock owners (n=30) were chosen by local Veterinarian. The preference to the flock owner was given whose birds were in egg laying condition. The average age of the birds was 24-25 weeks. If an owner agreed to participate, an in-person interview was conducted with the help of local Veterinarian in June 2013. At the time of the interview the written questionnaire was filled up by the interviewer and the interviews were recorded with an audio recorder (Sony, India). After the interview the biosecurity strategy which should be adopted to follow was ­conveyed in a farmers gathering with the help of local Veterinarian. After 3 months (September 2013) the place was revisited and the interview with the same questionnaire was conducted to the same flock owners interviewed earlier to detect their adoption of biosecurity practices. It was instructed to the flock owners to keep the egg laying records of their backyard hen and similar feed and other conditions should be maintained during the study period. The average day light hour during the study period was 13 h 17 min. As the study period was 3 months (June-August) only, no significant variation in day light hour was detected.

### Statistical analysis

The t-test was performed to detect the significant difference between the egg production level before and after adoption of biosecurity strategy in terai zone in SPSS version 21 (SPSS Inc., Chicago, IL, USA).

## Results

### Assessment of adoption of the biosecurity strategy

The adoption of the formulated strategy with consequences was measured in terai zone. The ‘terai’ zone was selected due to better interest and responsiveness of the farmers. Before adoption of the recommended strategy a low level of awareness demonstrated by the owners in terai zone about the biosecurity practices such as preparation of feed with boiled water (3%), cleaning frequency of feeding utensils and drinking trough (0% daily/weekly), frequency of change of drinking water in the trough (3.3% weekly), frequency of change of litter (0% daily), disposal of carcass by garden burial, washing of eggs (6%) and storage of eggs in room temperature (93%) (Table-1). In the studied area scavenging of the birds was mostly observed near the house (93%). The other birds such as crows (93%) and wild birds (6%) were observed in the studied area. The restriction of the visitors to the backyard premises was followed in the studied zone.

**Table-1:** Assessment of biosecurity practices by the backyard flock owners before and after adaptation of the recommended strategy in terai agro-climatic zone, West Bengal

Biosecurity measures (n=30)	Response before adaptation				Response after adaptation			
Preparation of feed with boiled water	1^a^ (3.33%)				4^a^ (13.33%)			
Cleaning frequency of feeding utensils and drinking trough	Daily/weekly	15 Days	Monthly	Any other	Daily	15 days	Monthly	Any other
	0	3 (10%)	27 (90%)	0	6 (20%)	9 (30%)	15 (50%)	0
Frequency of change of drinking water in the trough	Daily/weekly	15 Days	Monthly	Any other	Daily	15 days	Monthly	Any other
	1^a^ (3.33%)	27 (90%)	2 (6.6%)	0	18^a^ (60%)	10 (33.33%)	2 (6.66%)	0
Frequency of change of litter	Daily/weekly	15 Days	Monthly	Any other	Daily	15 days	Monthly	Any other
	0	27 (90%)	3 (10%)	0	18 (60%)	10 (33.33%)	2 (6.66%)	0
Washing of hands before providing feed to the birds	Yes		No		Yes		No	
	12* (40%)		18 (60%)		17* (56.66%)		13 (43.33%)	
Washing of hands and feet before entry or exit of the poultry houses	Yes		No		Yes		No	
	12* (40%)		18 (60%)		17* (56.66%)		13 (43.33%)	
Scavenging during daytime	Near house	Near paddy field	Near broiler farm	Any other	Near house	Near paddy field	Near broiler farm	Any other
	28 (93.33%)	2 (6.66%)	0	0	28 (93.33%)	2 (6.66%)	0	0
Presence of big tree near scavenging area	Yes		No		Yes		No	
	18 (60%)		12 (40%)		17 (56.66%)		13 (43.33%)	
Other birds observed in the flock premises	Duck	Pigeon	Crow	Wild birds	Duck	Pigeon	Crow	Wild birds
	0	0	28 (93.33%)	2 (6.66%)	0	0	28 (93.33%)	2 (6.66%)
Travelling of farmers to animal gatherings	Poultry market	Poultry show	Broiler farm	Other animal holdings	Poultry market	Poultry show	Broiler farm	Other animal holdings
	27 (90%)	0	0	3 (10%)	27 (90%)	0	0	3 (10%)
People involved in broiler farming entering the flock premises	Yes		No		Yes		No	
	2 (6.66%)		28 (93.33%)		1 (3.33%)		29 (96.66%)	
Disposal of carcass	Incineration	Garden burial	Feed to flock	Human consumption	Incineration	Garden burial	Feed to flock	Human consumption
	0	28 (93.33%)	0	2 (6.66%)	0	30 (100%)	0	0
Washing of eggs	Yes		No		Yes		No	
	2^a^ (6.66%)		28 (93.33%)		12^a^ (40%)		18 (60%)	
Preservation of eggs	Room temperature	4°C		Any other	Room temperature	4°C		Any other
	28 (93.33%)	2 (6.66%)		0	28 (93.33%)	2 (6.66%)		0
Vaccination	Ranikhet disease	Fowl pox		Any other	Ranikhet disease	Fowl pox		Any other
	28 (93.33%)	27 (90%)		0	28 (93.33%)	27 (90%)		0
Treatment with antibiotic	Yes		No		Yes		No	
	0		30 (100%)		0		30 (100%)	

a differs significantly at p<0.01,

* differs significantly at p<0.05

After providing the biosecurity strategy to the backyard owners of terai region the change in practices were noted in preparation of feed with boiled water (3.3% before adoption and 13.3% after adoption), cleaning frequency of feeding utensils and drinking trough (0% daily/weekly before adoption and 20% daily after adoption), frequency of change of drinking water in the trough (3.3% weekly before adoption and 60% daily after adoption), frequency of change of litter (0% daily before adoption and 60% daily after adoption), disposal of carcass by garden burial (93.3% before adoption and 100% after adoption), and washing of eggs (6.6% before adoption and 60% after adoption) (Table-1).

### Egg production

The average egg production/flock was increased significantly (p<0.01) in 3 months after adoption of the strategy comparing the last 3 months’ production before adoption, while other management practices and location remained same (Table-2).

**Table-2:** Number of eggs produced by the backyard birds before and after adoption of biosecurity strategy in terai zone, West Bengal.

Flock No	Flock size	Egg production before adoption (number of eggs/flock in last 3 months)	Egg production after adoption (number of eggs/flock in 3 months)
1	10	390	480
2	12	444	576
3	9	315	405
4	16	624	768
5	13	494	624
6	17	629	816
7	11	429	506
8	10	370	480
9	12	468	576
10	13	507	611
11	19	722	912
12	20	780	960
13	07	273	336
14	11	407	528
15	17	663	816
16	12	468	552
17	8	280	384
18	5	195	230
19	15	555	720
20	12	468	576
21	17	663	799
22	19	741	912
23	20	700	960
24	6	234	270
25	5	195	240
26	15	570	720
27	14	546	644
28	16	624	720
29	16	560	768
30	14	546	658
Total		495.3±30.00^a^	618.2±37.77^a^

a differs significantly (p<0.01)

## Discussion

The present study detected a low level of awareness demonstrated by the studied owners about the biosecurity practices. Similarly, in other developing countries such as Cambodia the mean frequency of yard cleaning per month and household was low [15]. In Bangladesh majority of the owners throw the carcasses of their backyard flocks in the nearby water bodies rather than burial and slaughter/sell the sick birds [16]. In Nigeria, different age groups of backyard birds are kept together [8]. In Myanmar, fencing of houses is not practiced to restrict the movement of the birds as more amount of supplementary feed is needed to maintain the flocks [17]. Further, disinfection of materials associated with backyard flocks, people and building is not followed in developing countries as reported earlier [9,10,18]. In contrast, in developed country such as in UK, moderately high level of awareness regarding daily cleaning frequency (20%), garden burial of the carcass, washing of eggs (60%) was detected among the backyard farmers [19]. In United States, indoor raising of the backyard birds with proper sanitary and other biosecurity measures are commonly practiced [20,21]. However, in developing countries it is not recommended because it increases the exposure of the owners to the zoonotic infection due to lack of biosecurity knowledge [8]. So the differences in education level, and culture explained the disparity in the biosecurity awareness. Although lack of knowledge about chicken infection (except Avian Influenza), reluctance to take Veterinary help during illness as observed in the present study was also detected among the backyard flock owners even in developed countries such as USA [22] and UK [23]. However, primary knowledge of Avian Influenza was detected in 40% responders which is in consistent with the earlier finding at Hooghly district (West Bengal) where 48% responders opined that the virus can affect both man and poultry [18].

In the studied area scavenging of the birds was mostly observed near the house (93%) which was considered as a good management practice because roaming in or near water lands may cause the birds being exposed to Avian Influenza or Ranikhet disease virus-infected wild birds or contaminated environments [4]. Specially the crows (93%) and wild birds (6%) were observed in the studied area. The restriction of the visitors to the poultry house (night poultry shelters) was followed in the studied zone which is similar with earlier findings [23]. However, majority of the owners (93%) did not wash their eggs after collection and stored them in room temperature which raised serious concern regarding food safety as this kind of practices helped to disseminate the pathogens within the contents of the eggs [24]. The *E. coli* was detected to penetrate the egg shell more efficiently with the increase in storage temperature of the eggs and penetration of *E. coli* paved the way for other Gram-positive bacteria such as *Staphylococcus* [25]. Therefore, it was recommended to keep eggs at cooling temperature without fluctuation.

The formulated biosecurity strategy was moderately well adopted among the farmers. Earlier several guidelines have been issued to train farmers on how to increase biosecurity in backyard flocks but a significant proportion of villagers continue their at-risk practices as observed in several countries [26,27]. This discrepancy was explained by the fact that measures were often costly and may not be correlated with the economic benefits of the farmers [28]. In Bangladesh also the biosecurity recommendation issued by the Government to decrease the transmission of Avian Influenza was not followed by the farmers due to change in practices caused financial losses [16]. Whereas, the present study has recommended a relatively cheaper strategy comprising the cleaning with ash/common detergent powder instead of costly disinfectants, sprinkle of detergent water left after washing of clothes in the scavenging area etc. So the strategy was moderately well adopted among the farmers in the test zone. However, two parameters of the formulated biosecurity strategy were not adopted by the farmers such as restricted travelling of the farmers to the local poultry market and preservation of eggs in cold temperature. Probably a single meeting for conveying the strategy to the farmers was not sufficient to make them understand.

The average egg production/flock was increased significantly (p<0.01) in 3 months after adoption of the strategy comparing the last 3 months’ production before adoption, while other management practices and location remained same. Similarly, Fasina *et al*. [29] demonstrated that the adoption of biosecurity strategy in backyard poultry sector in Egypt significantly increased the net income of the farmers.

## Conclusion

Thus the present study detected the implementation of the biosecurity strategy in backyard poultry farming in West Bengal can substantially benefit the farmers in terms of increased egg production. So the biosecurity strategy developed under the present study is recommended to implement in the backyard poultry for better and hygienic production.

## Author’s Contribution

SNJ, DG and PKD designed the study. IS conducted the study, drafted and revised the manuscript. US had done statistical analysis. All authors read and approved the final manuscript.

## References

[1] FAO (2000). Statistical database of Food and Agricultural Organization of the United Nations, Rome, Italy.

[2] Ahuja V, Dhawan M, Punjabi M, Maarse L (2008). Economics of village poultry.

[3] Pica-Ciamarra U, Otte J (2009). Poultry, Food Security and Poverty in India:Looking Beyond the Farm-Gate. Pro-Poor Livestock Policy Initiative (RR Nr. 09-02), FAO.

[4] Conan A, Goutard F.L, Sorn S, Vong S (2012). Biosecurity measures for backyard poultry in developing countries:A systematic review. BMC Vet. Res.

[5] Newell D.G, Elvers K.T, Dopfer D, Hansson I, Jones P, James S, Gittins J, Stern N.J, Davies R, Connerton I, Pearson D, Salvat G, Allen V.M (2011). Biosecurity-based interventions and strategies to reduce *Campylobacter* spp. on poultry farms. Appl. Environ. Microbiol.

[6] White P.L, Baker A.R, James W.O (1997). Strategies to control *Salmonella* and *Campylobacter in* raw poultry products. Rev. Sci. Tech.

[7] Siekkinen K.M, Heikkila J, Tammiranta N, Rosengren H (2012). Measuring the costs of biosecurity on poultry farms:A case study in broiler production in Finland. Acta Vet. Scand.

[8] Alhaji N.B, Odetokun I.A (2011). Assessment of biosecurity measures against highly pathogenic avian influenza risks in small-scale commercial farms and free-range poultry flocks in the North central Nigeria. Transbound. Emerg. Dis.

[9] Hamilton-West C, Rojas H, Pinto J, Orozco J, Herve-Claude L. P, Urcelay S (2012). Characterization of backyard poultry production systems and disease risk in the central zone of Chile. Res. Vet. Sci.

[10] Lukman D, Ridwan Y, Wibowo B, Basri C, Sudarnika E, Sugama A, Hermans P, Nell A (2011). Biosecurity practices in village poultry in Cipunagara Subdistrict, Subang District, West Java:Case study. In:Proceedings of 1^st^ International Congress of South East Asia Veterinary School Association, July 20-22, 2011, Bogor, Indonesia.

[11] Smith E.I, Reif J.S, Hill A.E, Slota K.E, iller R.S, Bjork K.E, Pabilonia K.L (2012). Epidemiologic characterization of colorado backyard bird flocks. Avian Dis.

[12] Annual Administrative Report (2009-10). Animal Resources and Development Department, Government of West Bengal.

[13] Samanta I, Joardar S.N, Das P.K, Das P, Sar T.K, Dutta T.K, Batabyal S, Isore D.P (2014). Virulence repertoire, characterization and ntibiotic resistance pattern analysis of *Escherichia coli* isolated from backyard layers and their environment in India. Avian Dis.

[14] Samanta I, Joardar S.N, Das P.K, Sar T.K, Bandyopadhyay S, Dutta T. K, Sarkar U (2014a). Prevalence and antibiotic resistance profiles of *Salmonella* serotypes isolated from backyard poultry flocks in West Bengal. India. J. Appl. Poult. Res.

[15] Conan A, Ponsich A, Goutard1 F, Khiev R, Tarantola A, Sorn S, Vong S (2013). A community-based education trial to improve backyard poultry biosecurity in rural Cambodia. Acta Trop.

[16] Sultana R, Rimi N. A, Azad S, Islam M. S, Khan M. S, Gurley E. S, Nahar N, Luby S. P (2012). Bangladeshi backyard poultry raisers’ perceptions and practices related to zoonotic transmission of avian influenza. J. Infect. Dev. Ctries.

[17] Henning J, Khin A, Hla T, Meers J (2006). Husbandry and trade of indigenous chickens in Myanmar–results of a participatory rural appraisal in the Yangon and the Mandalay divisions. Trop. Anim. Health Prod.

[18] Datta S, Sen S, Sengupta B (2010). A study on knowledge and practice related to bird flu in a rural community of Hooghly District of West Bengal. Indian J. Public Health.

[19] Karabozhilova I, Wieland B, Alonso S, Salonen L, Häsler B (2012). Backyard chicken keeping in the Greater London Urban Area:Welfare status, biosecurity and disease control issues. Br. Poult. Sci.

[20] Garber L, Hill G, Rodriguez J, Gregory G, Voelker L (2007). Non-commercial poultry industry:Surveys of backyard and gamefowl breeder flocks in the United States. Prev. Vet. Med.

[21] Yendell S.J, Rubinoff I, Lauer D.C, Bender J.B, Scheftel J.M (2012). Antibody prevalence of low-pathogenicity avian influenza and evaluation of management practices in Minnesota backyard poultry flocks. Zoonoses Public Health.

[22] DEFRA (2006). The Structure of the United Kingdom Poultry Industry. Hobby and ‘fancy poultry sector’.

[23] Burns T.E, Kelton D, Ribble C, Stephen C (2011). Preliminary investigation of bird and human movements and disease-management practices in non commercial poultry flocks in south western British Columbia. Avian Dis.

[24] Hutchison M.L, Gittins J, Walker A, Sparks N, Humphrey T.J, Burton C, Moore A (2003). An assessment of the microbiological risk involved with egg washing under commercial conditions. J. Food Prot.

[25] Al-Natour M.Q, Alaboudi A.R, Al-Hatamelh N.A, Osaili T.M (2012). *Escherichia coli* O157:H7 facilitates the penetration of *Staphylococcus aureus* into table eggs. J. Food Sci.

[26] FAO (2005). Prevention and Control of Avian Flu in Small Scale Poultry. A Guide for Veterinary Paraprofessionals in Vietnam.

[27] FAO (2006). Guide for the Prevention and Control of Avian Flu in Small Scale Poultry.

[28] Aini I (2000). Biosecurity in family flocks. Proceedings of the 21^st^ World’s Poultry Congress, 20–24 August, Montreal, Canada.

[29] Fasina F.O, Ali A.M, Yilma J.M, Thieme O, Ankers P (2012). The cost-benefit of biosecurity measures on infectious diseases in the Egyptian household poultry. Prev. Vet. Med.

